# Growth Performance and Nutritional Content of Tropical House Cricket (*Gryllodes sigillatus* (Walker, 1969)) Reared on Diets Formulated from Weeds and Agro By-Products

**DOI:** 10.3390/insects16060600

**Published:** 2025-06-06

**Authors:** Henlay J. O. Magara, Sylvain Hugel, Brian L. Fisher

**Affiliations:** 1 Department of Feed Development, Madagascar Biodiversity Center, Antananarivo 101, Madagascar; hugels@inci-cnrs.unistra.fr (S.H.); bfisher@calacademy.org (B.L.F.); 2 Institut des Neurosciences Cellulaires et Intégratives, UPR 3212 CNRS-Université de Strasbourg, 67087 Strasbourg, France; 3 Department of Entomology, California Academy of Sciences, 55 Music Concourse Drive, San Francisco, CA 94118, USA

**Keywords:** *Gryllodes sigillatus*, entomophagy, diets, weeds and agro by-products, nutrition analysis

## Abstract

The tropical house cricket (*Gryllodes sigillatus*) can convert organic diets formulated from weeds and agro by-products into high-quality biomass. This study assessed the potential of diets developed from weeds and agro by-products as a feed source for *G. sigillatus*. We compared the development and nutritional value of crickets fed these alternative diets with control crickets fed chicken feed. Ten different diets with varying protein contents were used, including chicken feed (Control) with a protein content of 215 g/Kg dry matter (DM) basis), Cassava–Sugar Diet (250 g/Kg DM protein) Desmodium–Bran Diet (245 g/Kg DM protein), Morning Glory–Bean Diet (240 g/Kg DM protein), Morning Glory–Cassava Diet (235 g/Kg DM protein), Morning Glory–Cowpea Diet (225 g/Kg DM protein), Mixed Weed–Bran Diet (Optimal) (215 g/Kg DM protein) Cassava–Gallant Soldier Diet (200 g/Kg DM protein), Wheat–Bran Diet (145 g/Kg DM protein), and Maize–Cassava Diet (135 g/Kg DM protein). The weight and length of the crickets were measured for 9 weeks from day 1 after hatching to day 56. Then, the crickets were harvested and analyzed for dry matter, crude protein, fat, ash, fiber, minerals, and fatty acid composition. Cricket developmental time, survival rate, weight and length, yield, proximate components, and mineral and fatty acids differed depending on the diet provided. The Mixed Weed–Bran Diet (Optimal) resulted in the crickets developing faster (48.8 days), with a higher survival rate (88.1%), greater adult length (19.2 cm) and weight (0.44 g), and a nutrition content richer in minerals and unsaturated fatty acids when compared to other treatments. Oleic, linoleic, and palmitic acids were the major fatty acids. The highest protein content (64.4 g/100 g) was observed in the Mixed Weed–Bran Diet (Optimal) and Morning Glory–Cassava Diet treatments, while the Maize–Cassava Diet treatment crickets possessed the highest quantities of fats (19.1 g/100 g) and ash (15.4 g/100 g). The fatty acid profile of *G. sigillatus* revealed the cricket to have high unsaturated fatty acids except in crickets fed Morning Glory–Cowpea Diet and Wheat–Bran Diet. Generally, *G. sigillatus* grew best and had the most nutritious body composition on the Mixed Weed–Bran Diet (Optimal). The findings indicate that diets developed from weeds and agro by-products have great potential to be used as an alternative feed source for crickets and are capable of replacing expensive chicken feed, enhancing the circular farming potential of insect farming.

## Simple Summary

*Gryllodes sigillatus* is a key edible cricket that possesses a significant nutritional benefit and excellent feed conversion ratio (FCR) and requires less feed to produce protein compared to traditional livestock like cattle or poultry. Despite the potential of insects in sustainable food systems, their role in managing agro-waste and weeds through rearing remains underexplored, and empirical research on their ecological interactions and waste conversion efficiency is limited. This research addresses critical research gaps and priorities to advance the integration of insects into sustainable food systems in Madagascar. This study evaluated the impact of formulated diets from weeds and agro by-products on growth parameters of *G. sigillatus* nymphs in Madagascar. Additionally, it determined the nutritional composition of crickets fed the developed diets. The results revealed that the diets offered to the crickets significantly affected the growth parameters and nutritional value of the adult insects in different ways. These findings suggest that weeds and agro by-products may be used to formulate inexpensive, accessible, and sustainable cricket diets that can replace chicken feed. The resulting crickets can be used to reduce malnutrition in Madagascar while facilitating the management of weeds and agro by-products as part of a circular farming system.

## 1. Introduction

The global human population is predicted to hit the 10-billion mark by 2050, with most of this growth expected to be in the Global South [1,2]. To meet the food demands of this growing population, food production must increase by over 70% between now and 2050 [3,4,5]. But increasing food production in the Global South will be complicated by limitations in the amount of arable land, energy, and water, among other factors [6,7,8]. Traditional food production systems often result in negative effects on the environment, such as clearing forests for farm fields, destruction of soils due to continuous farming, and emissions of carbon dioxide, water vapor, methane, and ammonia gases [9]. Alternative food sources are sorely needed to counter these challenges. One such alternative is farmed edible crickets. Crickets are rich sources of nutrients for human consumption and animal feed, such as proteins, lipids, minerals, vitamins, energy, fatty acids, and amino acids [10,11,12]. Edible crickets have many benefits which make them more sustainable animals to farm. These include crickets’ ability to consume organic by-products from both agricultural and industrial sources, as well as having less negative impacts on the environment than traditional livestock species, high feed conversion ratios, and higher egg-laying capacity with a short life period, requiring far less space and little water for production, and emitting far fewer greenhouse gases than livestock [13,14,15,16]. In addition, the organic waste produced from farming crickets, known as frass, is an effective crop fertilizer [17].

The culture of consuming various species of cricket dates back to historic times and is a habit reported in various anecdotes [18,19,20]. Over 60 species of edible cricket have been reported to be consumed globally [11]. These crickets are consumed as either food or traditional medicine and are also used as feed for poultry and fish by over two billion people globally, especially in Africa, Asia, and South America [21,22,23,24].

One of the crickets consumed globally is the tropical house cricket (*Gryllodes sigillatus* Walker, Orthoptera: Gryllidae). *Gryllodes sigillatus is* a small brown cricket with two dark bands across its thorax. As its vernacular name suggests, this species is found in the tropics and subtropics [25]. Adult males have wings covering the body halfway, enabling them to emit quiet chirps, while females have inconspicuous wings [26]. This cricket is emerging as a promising alternative to *Acheta domesticus*, *Pteronemobius nigrovus, Teleogryllus commodus* and *Teleogryllus oceanicus* for farming because of its high adaptability to many ecological zones, fairly herbivorous nature, low propensity to bite, more neutral smell and low propensity to be cannibalistic compared to other edible crickets [26,27,28]. Additionally, *G. sigillatus* demonstrates a high feed conversion rate and has the capacity for large-scale production as food and feed [29,30]. Recently, *G. sigillatus* meal has been successfully used to supplement human food and for poultry and fish feed [29,30].

Despite *G. sigillatus* being recognized as a promising future food resource, its sustainable and low-cost production remains limited due to insufficient research on the suitability of agricultural by-products as insect feed, as well as its ecological and economic impacts within a circular farming system.

Transitioning to organic waste streams such as weeds and agro by-products from organic farms could reduce environmental impacts, enhance resource efficiency, and support sustainable food production. *Gryllodes sigillatus* can be fed organic waste streams, resulting in nymphs and adults having excellent biomass and serving as good sources of protein, fats, fiber, ash, minerals and fatty acids [29]. The biomass and nutrient content of *G. sigillatus* can reach high levels on a dry matter basis, depending on the diet provided [10,15,19]. Previously, this cricket has been reared on chicken feed, which is expensive for the farmers to afford [25]. Moreover, this cricket has been reported to be farmed on single agro by-products, industrial food wastes, and a mixture of agro and industrial by-products [14,30,31]. However, no study has explored raising this cricket on diets developed from a combination of weeds and agro by-products. In this context, weeds are primarily unwanted plants growing where they are not required [8]. On the other hand, agro by-products are low-value agri-food wastes generated during various stages of agricultural and food processing activities [14,32]. Weeds and agro by-products possess several nutritional, functional, and bioactive components which can be used to develop innovative feed products, reduce waste, and improve the overall sustainability of cricket farming globally [32,33,34]. The selected weeds for the development of experimental diets against *G. sigillatus* include silver leaf *Desmodium* (*Desmodium uncinatum* (Jacq.) DC.), tropical African morning glory (*Ipomoea alba* L.), and gallant soldier (*Galinsoga parviflora* Cav.). On the other hand, the selected agro by-products included cassava leaves (*Manihot esculenta* L.), wheat bran (*Triticum aestivum* L.), maize bran (*Zea maize* L.), cowpea bran (*Vigna unguiculata* (L.) Walp), navy bean bran (*Phaseolus vulgaris*), maize germ (Zea mays L.), rice bran (*Oryza sativa* L.), taro leaves (*Colocasia esculenta* (L.) Schott), and cassava tuber bran (*Manihot esculenta* L.). These weeds and agro by-products were selected for the study because they are reported to be accepted as feed by farmed crickets [8]. Moreover, they are locally available in high abundance [8]. At the same time, these weeds have been reported to be less expensive than the chicken feed usually used by cricket farmers [8]. Additionally, they are said to be rich in nutrients to support the life cycle of farmed crickets [8].

The objective of the present study was to compare the suitability of certain diets formulated from weeds and agro by-products, including chicken feed (control), Cassava–Sugar Diet, Desmodium–Bran Diet, Morning Glory–Bean Diet, Morning Glory–Cassava Diet, Morning Glory–Cowpea Diet, Mixed Weed–Bran Diet (Optimal), Wheat–Bran Diet, and Maize–Cassava Diet for feeding *G. sigillatus*. We tested their effects on the growth rate and nutritional composition of *G. sigillatus*. Lastly, we discuss the implications of our findings for the effective mass farming of G. sigillatus in Madagascar and beyond.

## 2. Materials and Methods

### 2.1. Gryllodes sigillatus Colony

*Gryllodes sigillatus* eggs were collected from a colony established in April 2021 and maintained in the Madagascar Biodiversity Center Entomology Laboratory in Antananarivo, Madagascar, at a density of 0.3 crickets/cm^3^ for adult crickets. The source population of adult male and female crickets for starting the mother colony was collected from suburbanized areas around Antananarivo, Madagascar. The eggs for the experiment were obtained from a continuous mother colony of insects raised on chicken feed for two generations to amass a sufficient population for the experiments. Wet cotton wool balls were used to collect eggs from the colony. The eggs used in this study were less than 2 h old [35]. The cotton balls with the eggs were opened into sheets and placed in layers in a plastic container measuring 18.7 × 12.6 × 7.8 cm (Super 2; Aristoplast Products Pvt. Ltd., Mumbai, India). The eggs were dampened with water using a spraying hand pump. The containers were covered with lids and the ventilation holes overlaid with 0.2 mm wire gauze. The eggs were maintained in the laboratory at 30.0 ± 1.0 °C, 70.0% relative humidity, and a photoperiod of 12:12 (L: D) until hatching.

### 2.2. Cricket Feeding Experiments

The experiment utilized cleaned plastic containers measuring 18.7 × 12.6 × 7.8 cm for group cricket experiments (30 crickets per container). Although this density was low compared to regular rearing density, we selected it because it was more suitable for our growth study. This choice reduces stress in crickets, which could affect our results, diminishes cannibalism, and lowers competition for feed, allowing us to obtain more reliable data by subjecting the crickets to less environmental variation between replicates. The containers were covered with lids with openings measuring 17 × 10 cm fitted with 0.02 mm wire mesh for ventilation, to prevent crickets from escaping and to block the entry of predators. They were stored under the same environmental conditions as previously described. Diets were weighed and distributed to the rearing plastic containers at a uniform feeding rate of 100 mg/nymph every 3 days, amounting to 3 grams for 30 juveniles at the start of the experiment; however, this was adjusted to 5 grams as the crickets grew [36]. The uniform feeding rate of 100 mg/nymph every 3 days was determined by conducting a preliminary feeding experiment with 30 juveniles per box using chicken feed as a reference for 28 days before the actual tests for developed diets. At this feeding rate, the nymphs were found to leave some unconsumed diet. At any rate, below that, the nymphs consumed everything. During rearing, 30 one-day-old *G. sigillatus* nymphs were introduced per container, with each treatment having 10 replicates (n = 300). The diets were exchanged every three days, and clean tap water was replenished daily. The data was recorded weekly.

### 2.3. Diets and Their Preparation

The weeds, agro by-products, and other materials used in developing the nine different diets were obtained from organic farms and local sellers in Antananarivo. The weeds included silver leaf *Desmodium* (*Desmodium uncinatum* (Jacq.) DC.), tropical African morning glory (*Ipomoea alba* L.), and gallant soldier (*Galinsoga parviflora* Cav.). The agro by-products procured were cassava leaves (*Manihot esculenta* L.), wheat bran (*Triticum aestivum* L.), maize bran (Zea maize L.), cowpea bran (*Vigna unguiculata* (L.) Walp), navy bean bran (*Phaseolus vulgaris*), maize germ (Zea mays L.), rice bran (*Oryza sativa* L.), taro leaves (*Colocasia esculenta* (L.) Schott), and cassava tuber bran (*Manihot esculenta* L.). The other materials obtained from the sellers were white sugar made from sugar cane and baking powder. These ingredients were selected for the study because they are acceptable as feed by farmed crickets, locally available in high abundance, affordable to many cricket farmers compared to chicken feed and rich in protein content [8]. The silver leaf *Desmodium*, tropical African morning glory, gallant soldier, cassava leaves and taro leaves were cleaned using clean tap water, sun-dried for three days, and milled into single-ingredient powders used to develop the diets [12]. The study diets developed included (1) chicken feed (control); (2) cassava leaf powder, sugar and baking powder; (3) silver leaf *Desmodium*, wheat bran, maize bran, and cowpea bran; (4) tropical African morning glory powder, maize bran, navy bean bran, maize germ, and rice bran; (5) tropical African morning glory powder, cassava leaf powder, maize bran, and rice bran; (6) tropical African morning glory powder, cassava leaf powder, cowpea bran, and navy bean bran; (7) tropical African morning glory powder, gallant soldier powder, cassava leaf powder, cowpea bran, navy bean, maize bran, and wheat bran; (8) cassava leaf powder, gallant soldier powder, cowpea bran, tropical African morning glory powder, and taro leaf powder; (9) wheat bran and baking powder; and (10) maize bran, cassava tuber bran, wheat bran, and baking powder (Table 1).

### 2.4. Impact of Developed Diets and Chicken Feed on the Development and Survival Rate of Gryllodes sigillatus

To determine the impact of the developed diets and the chicken feed on the development time and survivorship of *G. sigillatus*, a group of 30 one-day-old nymphs were introduced into a ventilated plastic container forming a group to be offered one kind of developed diet. Each diet was replicated 10 times (n = 300). The total number of nymphs used for the 10 diets was 3000. At the start of the experiment, a group of *G. sigillatus* nymphs in a cage was provided with 3 g of food in a 4 cm diameter plastic Petri dish. As the nymphs grew, the feed quantity was increased to 5 g to meet their increased consumption needs. The 3 g provided at the start of the experiment and the 5 g diets offered later to cricket nymphs were considered sufficient to sustain the nymphs as they grew. The experimental nymphs were offered water ad libitum using a cotton sheet measuring 5 cm by 4 cm (length and width) dipped in water and spread in a rectangular plastic container with an internal length measuring 5 cm and a width of 4 cm. Then, the crickets in each box were provided three pieces of egg crates stacked on each other as a hideout. Each piece of egg crate measured 16 cm by 12 cm by 5 cm. All food not eaten was recovered from each box after three days and fresh food was introduced. Also, the cotton sheets used to supply water were withdrawn and fresh water was introduced in fresh cotton sheets. The cricket nymphs were checked daily until they reached adult stage [33]. Cricket development time was then calculated as the number of days between the hatching of the egg and the time of the emergence of the adult cricket [35]. The number of crickets alive at the end of the experiment, divided by the number of cricket nymphs at the start of the experiment and multiplied by 100, was the percent survivor rate for the crickets in each treatment [36].

### 2.5. Impact of Developed Diets and Chicken Diet on Wet Body Weight, Body Size and Yield of Gryllodes sigillatus

Nymph wet weights and sizes were recorded across all the replicates in all the feeding experiments. The measurement was performed once at the start of the experiment and repeated after every 7 days until the 56th day, when the experiment was stopped. During this time, 10 nymphs were randomly separated from each container into a transparent cup with the help of a fine camel’s hair brush for body weight and size measurement. Every feeding diet was replicated 10 times. Therefore, 100 insects (n = 100) were sampled from each diet. Considering all 10 feeding diets, 1000 nymphs (100/experimental diet) were randomly picked from experimental containers each sampling day. The sampled nymphs from each replicate were then put into a transparent plastic cup (7.5 cm diameter × 12.5 cm height) covered with a transparent Petri dish, and the total mass was measured using an analytical balance with a precision of 0.001 g (Kern and Sohn, Ballngen, Germany). The wet body mass of the cricket was recorded as the mass of the transparent Petri dish, and the mass of the transparent plastic cup was subtracted from the total mass [12,16]. The wet body size of each nymph was measured using a ruler with a precision of one millimeter [19]. In containers where high mortality resulted in fewer than 10 surviving crickets, the body weight and length of all the crickets were measured.

### 2.6. Impact of Developed Diets and Chicken Diet on Feed Conversion Ratio and Yield of Gryllodes sigillatus on a Simulated Small-Scale Farm

To determine the yield of *G. sigillatus* fed each developed diet and chicken feed, 2500 one-day-old nymphs were introduced into a rearing box with an internal length of 48 cm, width of 31 cm, and height of 27 cm with a ventilated lid. For each diet, there were five replicates (n = 5). The total number of crickets used per diet was 12,500. Cricket nymphs in each box were offered 5 g of diet in the first 14 days and the diets were adjusted to 10 g as the crickets increased in size and their consumption increased. The nymphs in each rearing box were also provided with water in cotton sheets placed uniformly in a shallow plastic container with rough walls to enable them to climb and reach the water. Then, the nymphs were given five egg crates as hideouts. The remaining food was removed from each box every three days and replaced with fresh food. Similarly, the cotton sheets were exchanged after three days [8]. The unconsumed food mixed with cricket feces recovered from each box was separated, dried, and weighed following the protocol of [12]. At the end of the experiment, the yield for each treatment was determined by weighing all crickets per container. The total output was then pooled to identify the most effective diet. Thus, the crickets were reared up to the 56th day. Then, the crickets from each rearing box were harvested into a transparent plastic container and weighed. The weight of the crickets in each container was obtained by subtracting the weight of the transparent plastic container from the weight of the crickets and the plastic container. The amount of feed consumed per group was determined by subtracting the dried unconsumed diet from the initial feed offered to the cricket at each feeding session. Then, the amount of food consumed in eight weeks (56 days) was pooled per replicate and used to calculate the feed conversion ratio (FCR) of crickets in each rearing container. The FCR was subsequently obtained by dividing the total diet consumed/total weight gain [37,38].

### 2.7. Gryllodes sigillatus Cricket Samples and Their Preparation

The same ratio of female to male adults of tropical house crickets reared on nine developed diets and chicken feed as the control were placed into Ziplock plastic bags. Then, the crickets were put into a deep freezer for five minutes to make them inactive. Next, the crickets were washed using tap water to remove any feed material or cricket droppings that could have escaped with the harvested crickets into the Ziplock bags. The cleaned crickets were then killed by placing them in a deep freezer at −20 °C [39]. The dead crickets were then removed from the deep freezer and allowed to thaw at room temperature. After thawing, the crickets were introduced in separate compartments of a convection oven (Memmert, Germany) set at 40 °C for 12 h to dry. The dried cricket samples were ground into a fine powder using a blender (MIKA MBLR2999WB, manufactured by SINSEEN, Cixi, China). The cricket powders were then stored in separate airtight bottles in the dark at −20 °C before being used for proximate, mineral element, and fatty acid analyses.

### 2.8. Proximate Content of Developed Diets, Chicken Feed and Gryllodes sigillatus Cricket Powders

Powders of *G. sigillatus* fed on developed diets and chicken feed were analyzed for proximate components following the Association of Official Analytical Chemists (AOAC) [40] methods, as explained by [41]. The dry matter (DM) content of the samples was determined by drying the sample in a 105 °C oven for 3 h (Method No. 930.15) [42]. The Kjeldahl method was used to analyze the protein content in the developed diets, chicken feed and cricket samples fed on the developed diets. A nitrogen-to-protein conversion factor of 6.25 was used to determine the crude protein for the developed diets and chicken feed [43]. On the other hand, a nitrogen-to-protein conversion factor of 5.09 was used to approximate the crude protein in *G. sigillatus* reared on different diets and chicken feed according to [44]. The nitrogen conversion factor 5.09 was chosen to prevent overestimating crude protein concentration with the Kjeldahl method [45]. The crude fat content was evaluated following the Soxhlet method (Method No. 2003.05) [40]. The amount of ash was obtained by burning cricket and diet samples at 550 °C in a muffle furnace until a constant weight was attained following Method No. 930.05 [42]. The quantity of crude fiber was determined by acid digestion followed by alkaline digestion as per the Weende Method (962.09). Carbohydrates in the samples were obtained by subtracting the mass g/kg DM of crude protein, total fat, crude fiber, water and ash from 1000 g/kg DM of samples according to [46]. Gross energy in crickets and diets was assessed using an adiabatic calorimetric bomb (Parr 1261; PARR Instrument, Moline, IL, USA) [2]. The analyses were performed in triplicates (n = 3) at the Nutrition Laboratory of International Livestock Research Institute, Nairobi, Kenya.

### 2.9. Mineral Element Analysis

The contents of iron (Fe), copper (Cu), zinc (Zn), manganese (Mn), sodium (Na), magnesium (Mg), calcium (Ca), phosphorus (P) and potassium (K) in the developed diets, chicken feed and tropical house cricket powders were analyzed using an atomic absorption spectrometer (Optima 4300™ DV ICP-OES, Perkin Elmer, Wellesley, MA, USA) following the standard method detailed by [47]. The analyses were performed at the International Livestock Research Institute, Nairobi, Kenya. Briefly, about 8 mL of concentrated nitric acid (16.2 mol/L) (VWR Chemicals, Fontenaysous-Bois, France) and 2 mL of 9.8 mol/L hydrogen peroxide (Sigma-Aldrich, St. Louis, MO, USA) were introduced into 0.5 g of each sample in a round-bottom flask and allowed to digest overnight in a fume chamber. After overnight digestion, the digested samples were further digested in a block digester (Model TE007-A, TECNAL, São Paulo, SP, Brazil) at 75 °C for 30 min, 120 °C for 20 min, 180 °C for 20 min, and 200 °C for 10 min. The formed wet ashes were allowed to cool in a fume hood. The cooled ashes were then dissolved in 0.4 mol/L nitric acid and diluted ten times to the right concentrations depending on the mineral element and the expected calibration curve. Standard solutions from certified stock solutions (Chem Lab, Zedelgem, Belgium) were diluted to give calibration standards of 400, 800, 2000, and 4000 g/L for each mineral element. The values of various mineral elements and the standard solutions were determined from an inductively coupled plasma–optical emission spectrometer (ICP-OES) (Optima 4300™ DV ICP-OES, Perkin Elmer, Wellesley, MA, USA). To set the external standards and record the data, Perkin Elmer Winlab 32 software (Perkin Elmer, USA) was employed [48]. The analyses were performed in triplicate.

### 2.10. Fatty Acid Profile

The fatty acids of the samples of tropical house crickets reared on different diets were extracted and analyzed according to the slightly modified protocol previously described by [49]. In summary, about 0.5 g of fat extracted from tropical house cricket samples was saponified with 100 µL dichloromethane and one milliliter of aqueous methanolic NaOH by refluxing for 10 min at 90 °C. Then, one milliliter of boron trifluoride–methanol (14%) was introduced into the solution and boiled in a water bath for 10 min. Next, 600 µL n-hexane was added to a saturated salt mixture to extract fatty acid methyl esters. Then, the upper layer was separated, and one microliter of the layer was injected into a Varian CP-3800 Gas Chromatograph (Varian, Walnut Creek, CA, USA) connected to a flame ionization detector (FID) chromatographed with hydrogen gas at a flow rate of 29 milliliters per minute, with a split ratio of 1 to 20. A CP-SIL capillary tube measuring 100 m × 0.25 mm, 0.2 µm (Varian Inc.), was used to separate the fatty acid methyl esters. The injector and detector were held at 300 °C and 280 °C, respectively. Nitrogen was the carrier gas during analysis. The temperature of the oven column was programmed at 70 °C and held for one minute before being increased at 5 °C/minute to 250 °C and held for 20 min. The methyl ester peaks were identified by their retention times and by comparing them with fatty acid methyl ester standards from Sigma-Aldrich, which were analyzed under the same conditions [50]. The fatty acid methyl esters were run in triplicate (n = 3).

### 2.11. Statistical Analysis

Statistical analyses were conducted using R version 4.4.2 [51]. A one-way analysis of variance (ANOVA) was used to evaluate differences in development time, survival rate, body weight, body length, feed conversion ratio, yield, proximate composition, mineral content, and fatty acid profiles. The Student–Newman–Keuls (SNK) test was employed as a post hoc procedure to identify significant differences between groups. Before performing ANOVA, assumptions of normality and homogeneity of variance were assessed using the Shapiro–Wilk test (*p* > 0.05) and Levene’s test (*p* > 0.05), respectively. Statistical significance was set at *p* < 0.05. The correlation between the chemical and mineral contents of the developed diets and chicken feed (control) on the cricket growth parameters was analyzed using principal component analysis (multivariate).

## 3. Results

### 3.1. Diet Composition

The contents of dry matter (DM), crude protein, crude fat, ash, crude fiber, carbohydrate, gross energy, iron (Fe), copper (Cu), zinc (Zn), manganese (Mn), sodium (Na), magnesium (Mg), calcium (Ca), phosphorus (P), and potassium (K) in the diets were recorded and are presented in Table 2 and Table 3. The DM across the diets ranged from 884.0 to 921.0 g/kg DM. The highest dry matter was recorded in the Mixed Weed–Bran Diet (Optimal) and the lowest in Desmodium–Bran Diet (Table 2). The crude protein concentration of the diets varied from 135 to 250 g/kg DM. The highest protein content was recorded in Cassava–Sugar Diet followed by Desmodium–Bran Diet, with the lowest in Maize–Cassava Diet (Table 2). The WBP diet was high in carbohydrates, while the carbohydrate content in SWMC was the lowest in g/kg DM. Fat was low in Maize–Cassava Diet and highest in Morning Glory–Bean Diet. Mixed Weed–Bran Diet (Optimal) had higher ash content, Fe, Zn, Mn and Na than other diets (Table 2 and Table 3). WBP had the lowest ash value (Table 2). Desmodium–Bran Diet had the largest quantity of fiber (227 g/kg DM), while the smallest amount was recorded in Morning Glory–Cowpea Diet (62 g/kg DM). A higher ash content was recorded from Mixed Weed–Bran Diet (Optimal) (118 g/kg DM) than other feeds. Mixed Weed–Bran Diet (Optimal) had the highest iron (881.4 mg/kg), zinc (64.2 mg/kg), manganese (79.3 mg/kg), and sodium (2399.9 mg/kg). Maize–Cassava Diet had the least amount of iron (38.4 mg/kg), copper (3.5 mg/kg), zinc (18.9 mg/kg), sodium (19.3 mg/kg), and macro-minerals and lower micro-mineral concentrations (Table 3). This data indicates that the nutritional composition of the diets varied significantly, with Mixed Weed–Brian Diet (Optimal) consistently showing the highest levels of essential nutrients, including crude protein, ash, and key minerals such as iron, zinc, manganese, and sodium, while Maize–Cassava Diet exhibited the lowest concentrations of these nutrients.

### 3.2. Impact of Tested Diets and Chicken Feed on the Development Time of Gryllodes sigillatus

**Figure 1:**
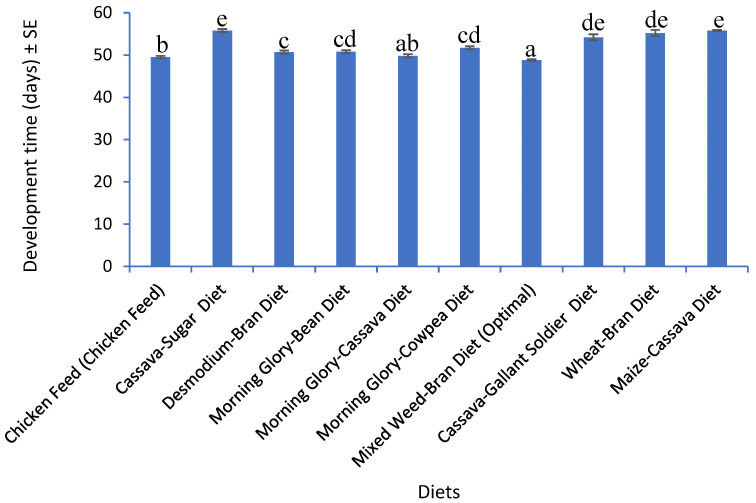
Mean (±SE) developmental time (days) of *Gryllodes sigillatus* (n = 10). Different superscripts per bar indicate significant differences, Student–Newman–Keuls test (*p* < 0.05).

*Gryllodes sigillatus* development time is a valuable parameter for measuring the influence of diet on farmed crickets. Development time of the nymphs was measured from hatching to eclosion of adult crickets. In this case, the developmental time of *G. sigillatus* fed different developed diets and chicken feed varied significantly (F_9,90_ = 42.52, *p* < 0.001) (Figure 1). Crickets fed Mixed Weed–Bran Diet (Optimal) reached adulthood in 48.8 ± 0.2 days, with development time measured as the number of days from hatching to the appearance of fully developed wings and adult morphology. The mean and standard error (±SE) were calculated from the recorded development times of 30 individuals per rearing box, monitored daily in controlled conditions (30 ± 1 °C, 70 ± 5% RH). Each individual was checked for molting and maturation indicators, and the standard error reflects variability across the population. On the other hand, crickets reared on chicken feed (control) reached adult stage on 49.5 ± 0.3 days. Crickets fed Cassava–Bran Diet and Maize–Cassava Diet had the longest developmental period at 55.8 ± 0.2 and 55.8 ± 0.1 days, respectively. This data indicated that Mixed Weed–Bran Diet (Optimal) resulted in the shortest developmental time, highlighting its potential for enhancing the growth rate of *G. sigillatus*.

### 3.3. Impact of Developed Diets and Chicken Feed on Survivorship of Gryllodes sigillatus

**Figure 2:**
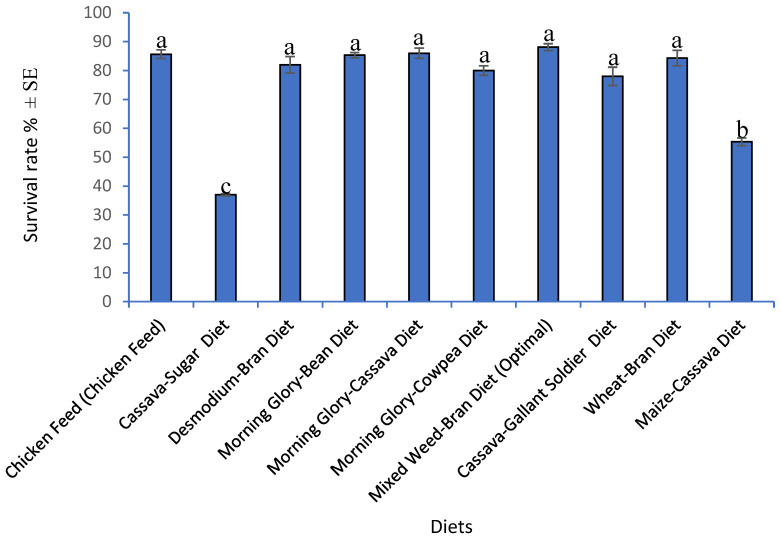
Mean (±SE) survival rate of *Gryllodes sigillatus* (n = 300) reared on different diets. Different superscripts per bar indicate significant differences, Student–Newman–Keuls test (*p* < 0.05).

The effect of feed on the survival rate of *G. sigillatus* fed different diets is presented in Figure 2. Survival rate was calculated as the percentage of individuals that reached adulthood from the initial number of hatched nymphs, under controlled rearing conditions. The developed diets and chicken feed significantly influenced the survival rate of *G. sigillatus* (F_9,90_ = 48.31, *p* < 0.001). Crickets fed Mixed Weed–Bran Diet (Optimal) recorded the highest survival rate (88.1%) compared to chicken feed (85.7%) and other developed diets. *G. sigillatus* grown on Cassava–Sugar Diet had the lowest survivorship (37%), demonstrating its poor suitability for promoting the survival of *G. sigillatus*.

### 3.4. Impact of Developed Diets and Chicken Feed on Wet Body Weight and Body Size of Gryllodes sigillatus

**Figure 3:**
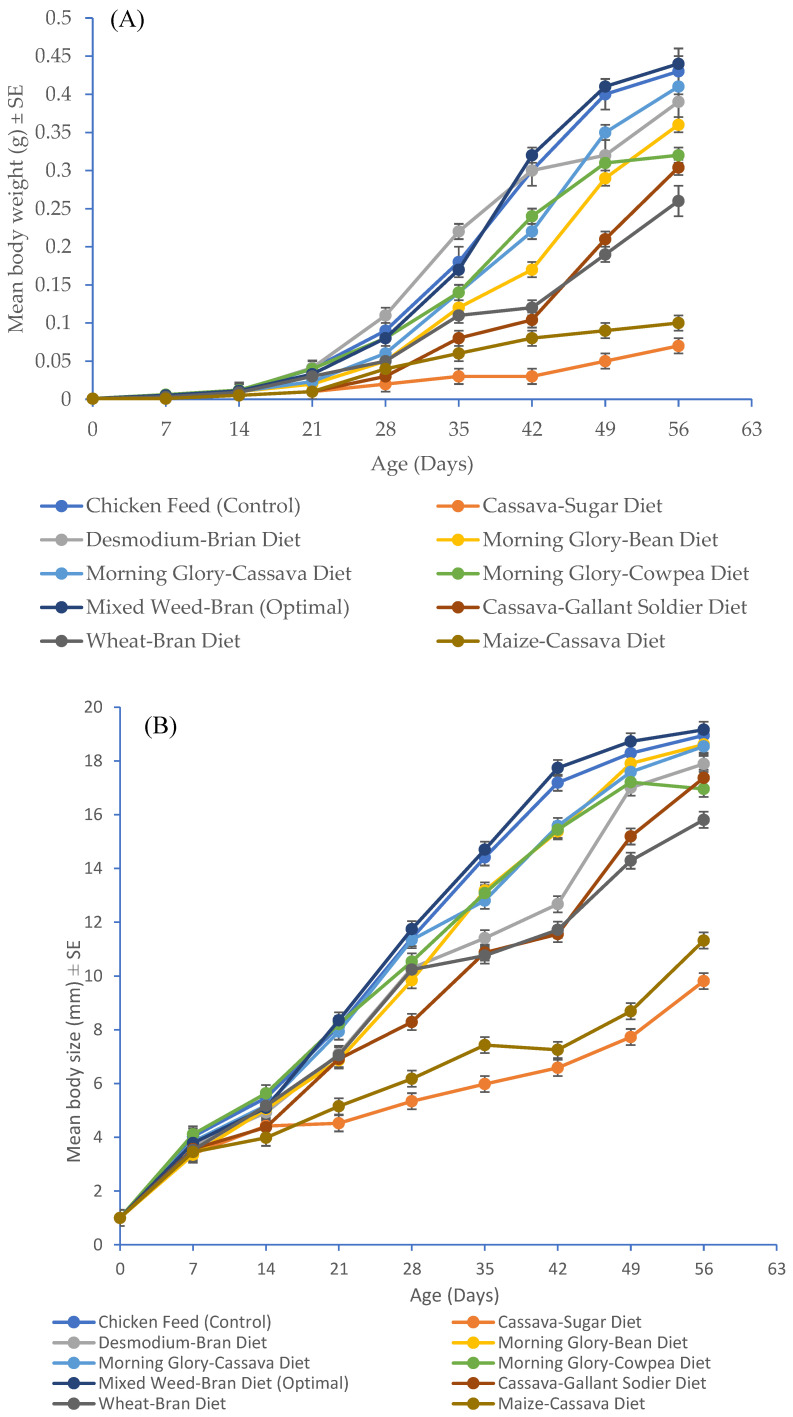
(**A**) Mean ± SE body weight and (**B**) mean ± SE body size of *Gryllodes sigillatus* (n = 100) reared on different diets.

Diets determine the yield output of farmed edible crickets by influencing cricket body weight and length. The body weight and size of the crickets grown on the developed diets and chicken feed are presented in Figure 3A,B. The body weight and size of the crickets on the last sampling day were significantly impacted by the ingredients of the developed diets and chicken feed (F_9,990_ = 328.00, *p* < 0.001; F_9,990_ = 94.49, *p* < 0.001, respectively). The highest average body weight (0.44 g) and body size (19.16 mm) were recorded in *G. sigillatus* crickets fed Mixed Weed–Bran Diet (Optimal) compared to crickets reared on chicken feed (0.43 g body weight and 18.95 mm body size), while those fed feed D2 had the lowest mean body mass (0.07 g) and body size (9.81 mm) (Figure 3A,B), indicating the superior effectiveness of Mixed Weed–Bran Diet (Optimal) in enhancing the yield of *G. sigillatus*.

### 3.5. Impact of Developed Diets and Chicken Diet on Feed Conversion Ratio and Yield of G. sigillatus on a Simulated Small-Scale Farm

**Figure 4:**
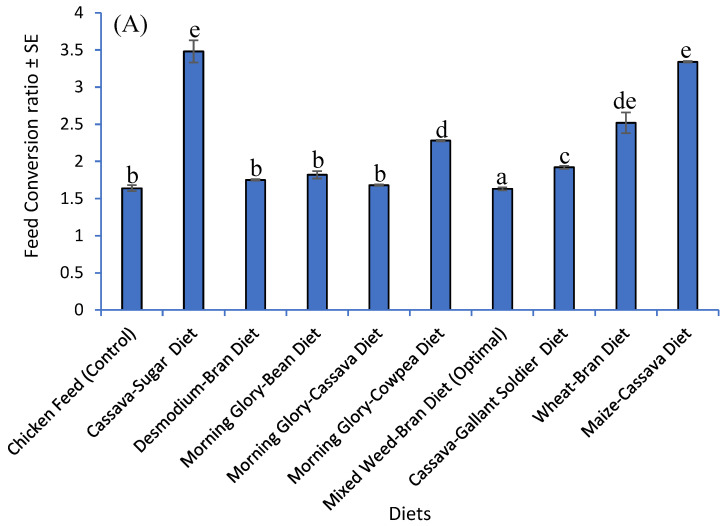

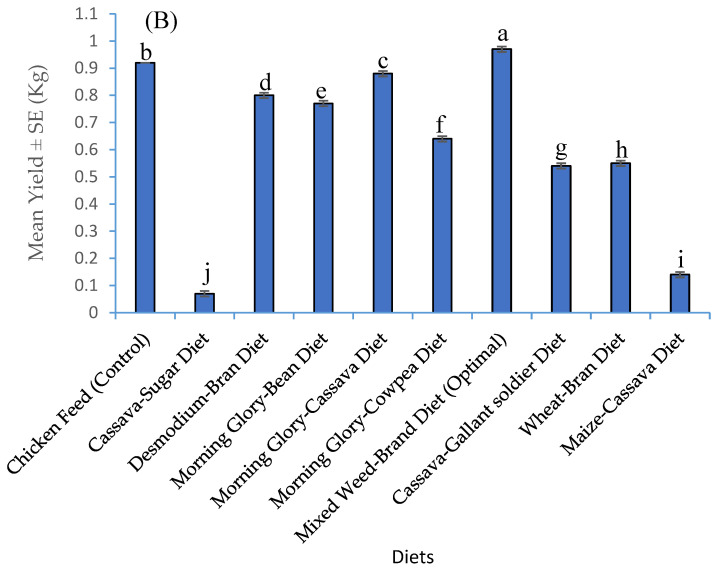
(**A**) Mean FCR (±SE) (n = 10) and (**B**) mean yield (±SE) of *Gryllodes sigillatus* (n = 5) reared on different diets. Different superscripts per bar indicate significant differences as calculated using the Student–Newman–Keuls test (*p* < 0.05).

The FCR and yield of the *G. sigillatus* reared on the developed diets and chicken feed are presented in Figure 4A,B. The FCR and yield of the *G. sigillatus* on the last sampling date were significantly influenced by the composition of the developed diets and chicken feed (F_9,90_ = 98.98, *p* < 0.001; F_5,45_ = 69,829.00, *p* < 0.001, respectively). The lowest FCR (1.63) was recorded for crickets fed the Mixed Weed–Bran Diet (Optimal) compared to chicken feed (control) (1.64) and other developed diets. The highest FCR (3.48) was recorded in *G. sigillatus* crickets fed Cassava–Sugar Diet and Maize–Cassava Diet (Figure 4A). On the other hand, the highest yield (0.97 Kg) was observed in crickets fed Mixed Weed–Bran Diet (Optimal), while the crickets fed chicken feed (control) had a yield of 0.92 Kg. The lowest yield was found among crickets reared on Cassava–Sugar Diet (Figure 4B). This data indicated that Mixed Weed–Bran Diet (Optimal) resulted in the most efficient feed conversion ratio and highest yield, demonstrating its potential for optimizing *G. sigillatus* production in small-scale farm settings.

### 3.6. Multivariate Analysis of Effect of Tested Diets and Chicken Feed Nutrition Content on Growth Performance Traits of Gryllodes sigillatus

**Figure 5:**
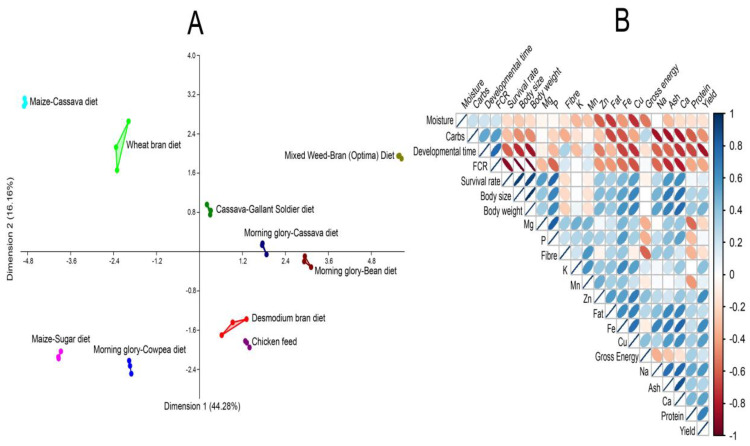
Multivariate analysis of diet effects on *Gryllodes sigillatus* growth performance. (**A**) Multidimensional scaling (MDS) plot showing the clustering of different diets based on their impact on key growth and nutritional traits. The Mixed Weed–Bran Diet (Optimal) is distinctly separated, indicating superior performance across multiple parameters. (**B**) Correlation matrix illustrating relationships among biological and nutritional traits. Blue ellipses indicate positive correlations; red ellipses represent negative correlations. Strong positive associations were observed between survival, yield, protein, ash, fat, and gross energy, while dietary moisture and developmental time were negatively correlated with performance outcomes.

As shown in Figure 5, the Mixed Weed–Bran (Optimal) Diet demonstrated the most favorable outcomes across all growth and nutritional parameters. This diet, containing 21.5% protein and high mineral content (ash), was distinctly separated from other diets in the multidimensional scaling (MDS) plot and was positively associated with high survival rate, yield, body weight, and feed conversion efficiency. Correlation analysis revealed that survival and performance were positively influenced by dietary protein, fat, ash, and gross energy and mineral elements, whereas higher dietary moisture content was negatively correlated with these traits, indicating that excessive moisture may dilute nutrient density. The Cassava–Gallant Soldier Diet and Morning Glory–Cassava Diet also showed moderate performance. In contrast, diets such as Maize–Sugar Diet, Morning Glory–Cowpea Diet, and chicken feed were clustered in the lower-performing region of the MDS plot and were associated with longer developmental times, higher FCR, and reduced yield and survival. These findings highlight the importance of nutrient-rich, well-balanced diets, particularly those high in protein and minerals, with controlled moisture levels for optimizing insect growth and development.

### 3.7. Proximate Composition

The contents of dry matter, crude protein, crude fat, ash, crude fiber, carbohydrate, and gross energy in the tropical house crickets fed on different diets are presented in Table 4. The diets offered to the crickets significantly impacted their proximate content (Table 4). The highest DM was recorded in adult *G. sigillatus* fed Mixed Weed–Bran Diet (Optimal), with the lowest in crickets fed Cassava–Sugar Diet. Adult cricket crude protein varied from 51.2 to 64.4 g/100 g DM and significantly differed among the crickets fed on developed diets and chicken feed. The *G. sigillatus* reared on Mixed Weed–Bran Diet (Optimal) had a similar protein content to those fed Morning Glory–Cassava Diet, with quantity being 64.4 g/100 g DM, respectively, and their protein value was higher than those reared on other developed diets (Table 4). The crude fat concentration varied between 7.4 and 19.1 g/100 g DM, reflecting significant variations among the diet treatments used to grow the crickets, with the highest fat and ash content found in *G. sigillatus* reared with D10. The lowest fat and ash were recorded in crickets fed Cassava–Sugar Diet and Wheat–Bran Diet, respectively. Meanwhile, adults of *G. sigillatus* fed the Cassava–Sugar Diet showed the highest amount of fiber, with figures of 10.3 g/100 g DM, whereas those fed Mixed Weed–Bran Diet (Optimal) exhibited the lowest amount of fiber (3.4 g/100 g DM). Crickets grown on Morning Glory–Cowpea Diet had the highest carbohydrate content (14.9 g/100 g DM) while those reared on Maize–Cassava Diet had the lowest. The gross energy of the crickets fed on different diets ranged from 3388.0 to 4242.0 kcal/kg DM. The highest gross energy was observed in crickets fed Mixed Weed–Bran Diet (Optimal), and the lowest was recorded from crickets fed Cassava–Sugar Diet (Table 4), indicating the superior nutritional profile of crickets reared on Mixed Weed–Bran Diet (Optimal) compared to other diets.

### 3.8. Mineral Elements in Gryllodes sigillatus Fed on Different Developed Diets and Chicken Feed

The values of Fe, Cu, Zn, Mn, Na, Mg, Ca, P, and K in the tropical house crickets fed different diets are recorded in Appendix A Table A1. The diets used to rear the crickets affected their elemental contents significantly (Table A1). Cricket Fe varied from 10.5 to 23.9 g/100 g DM depending on their diets. The crickets reared on Mixed Weed–Bran Diet (Optimal) had the highest Fe content (23.9 g/100 g DM) compared to crickets reared on other diets (Table A1). The lowest Fe was in crickets fed Desmodium–Bran Diet (10.5 g/100 g DM). The Mg content ranged between 95.1 and 172.1 g/100 g DM, showing significant differences among the treatments, with the highest Mg content found in crickets supplied with Cassava–Sugar Diet and the lowest in crickets fed with Maize–Cassava Diet. On the other hand, the crickets provided Mixed Weed–Bran Diet (Optimal) showed the highest amount of Ca, with a value of 839.1 g/100 g DM, while those fed with WBP had the lowest amount of Ca (183.6 g/100 g DM). The Cu in the cricket samples ranged from 0.21 to 4.5 g/100 g DM. Crickets grown on Cassava–Sugar Diet had more Cu than crickets reared on other diets and the control diet. The least Cu was detected in crickets reared on chicken feed (control). Phosphorus in the crickets ranged from 724.7 and 1495.7 g/100 g DM. Crickets raised on Mixed Weed–Bran Diet (Optimal) had a higher level of P, while lower P was recorded in crickets subjected to diet Maize–Cassava Diet. In the case of Zn, crickets provisioned with Mixed Weed–Bran Diet (Optimal) had the largest quantity (34.4 g/100 g DM), whereas those fed with Desmodium–Bran Diet had the least amount of Zn (19.4 g/100 g DM). The Mixed Weed–Bran Diet (Optimal) resulted in cricket samples with the highest K concentration (1607.4 g/100 g DM). The lowest K was observed in crickets given Maize–Cassava Diet as feed. The lowest Mn (4.9 g/100 g DM) was recorded in crickets cultivated using Desmodium–Bran Diet, while the highest Mn was noted in crickets provided with Cassava–Sugar Diet. The content of Na in reared crickets varied between 114.9 and 370.5 g/100 g DM. The highest Na level was recorded in crickets fed Mixed Weed–Bran Diet (Optimal) and the lowest was found in crickets fed Wheat–Bran Diet. This data indicated that Mixed Weed–Bran Diet (Optimal) resulted in crickets with the highest concentrations of several key minerals, including iron, calcium, phosphorus, zinc, and potassium, demonstrating the superior value of this diet to produce *G. sigillatus* of high mineral profile compared to the other diets tested.

### 3.9. Fatty Acid Content in Gryllodes sigillatus Crickets Reared on Different Developed Diets and a Control Diet

The fatty acid values of *G. sigillatus* raised on various developed diets and a control diet are presented in Table A2. Diets offered to the experimental *G. sigillatus* crickets significantly influenced the fatty acids of the respective crickets. *Gryllodes sigillatus* fed experimental diets had oleic acid levels ranging from 6.0 to 60.3% of total fats, with oleic acid being the major fatty acid in crickets fed both developed diets and the control. Oleic fatty acids were then followed by linoleic acid (9.23 to 30.1%), palmitic acid (0.5 to 37.1%), stearic acid (8.9 to18.5%), myristic acid (1.2 to 4.0%), arachidic acid (1.1 to 3.5%), margaric acid (0.7 to 2.1%), alpha-linolenic acid (0.8–1.5%), behenic acid (0.3 to 1.5%), lauric acid (0.1 to 0.9%), pentadecanoic acid (0.2 to 0.7%), and lignoceric acid (0.35%). Crickets fed Morning Glory–Cowpea Diet had the highest palmitic acid, stearic acid, myristic acid, margaric acid, and pentadecanoic acid concentrations compared to crickets reared on other developed diets and the control. Crickets exposed to Morning Glory–Bean Diet led to samples with the highest oleic acid, arachidic acid, behenic acid, and lauric acid compared to those fed the rest of the developed diets and the control. The highest linoleic acid, alpha-linolenic acid and lignoceric acid were recorded in crickets fed Cassava–Gallant Soldier Diet, while the crickets fed Maize–Cassava Diet had more margaric acid than those fed other diets. Cassava–Gallant Soldier Diet led to crickets with the highest essential fatty acids (EFAs) compared with the other diets. Crickets reared on Cassava–Gallant Soldier Diet had the highest ratio of polyunsaturated fatty acids (PUFAs) to saturated fatty acids, while the lowest PUFA ratio was recorded in crickets fed Maize–Cassava Diet. Crickets reared on chicken feed (control), Cassava–Sugar Diet, Desmodium–Bran Diet, Morning Glory–Bean Diet, Morning Glory–Cassava Diet, Mixed Weed–Bran Diet (Optimal), Cassava–Gallant Soldier Diet, and Wheat–Bran Diet resulted in crickets with higher quantities of unsaturated fatty acids. Lower amounts of unsaturated fatty acids were recorded in crickets offered Morning Glory–Cowpea Diet and Maize–Cassava Diet. This data indicated that diet Mixed Weed–Bran Diet (Optimal) resulted in crickets with the highest concentrations of polyunsaturated fatty acids (PUFAs) relative to saturated fatty acids, highlighting its potential to produce *G. sigillatus* with a more favorable fatty acid profile compared to other diets tested.

## 4. Discussion

Madagascar, renowned for its rich biodiversity, provides an abundant supply of weeds and agricultural by-products. These materials have traditionally been used as livestock feed, yet their potential as sustainable dietary inputs for tropical house crickets (*G. sigillatus*) remains largely untapped. This study aimed to evaluate the effectiveness of these low-cost resources in supporting cricket growth and nutritional value, with a broader goal of contributing to sustainable agricultural practices and circular farming systems.

Our findings demonstrate that *G. sigillatus* can thrive on diets formulated from locally available weeds and agro by-products. Notably, the Mixed Weed–Bran Diet (Optimal) and Morning Glory–Cassava Diet supported superior growth, survivorship, and nutrient accumulation. These diets significantly influenced cricket composition, especially dry matter, protein, fat, ash, and fiber content, confirming the critical role of diet in cricket development, as also noted in previous studies [8,14,52,53].

Crickets efficiently converted these diets into valuable biomass, reinforcing the feasibility of using regional plant-based resources in sustainable insect farming. Among all tested diets, the Mixed Weed–Bran Diet emerged as the most effective, enhancing growth rate, survival, body size, and nutritional composition. Its balanced nutrient profile—rich in protein, fats, carbohydrates, ash, gross energy, and essential minerals—likely accounts for its performance, as indicated by PCA analysis [36,54].

Protein is essential for insect tissue synthesis, molting, and hormone regulation. Diets with well-balanced amino acid profiles, particularly methionine and lysine, have been shown to accelerate growth and improve survival in related species such as *A. domesticus*, *G. bimaculatus*, and *G. madagascarensis* [36,39,54,55]. Carbohydrates serve as primary energy sources, and the protein-to-carbohydrate (P:C) ratio is a major determinant of growth efficiency. For instance, *G. bimaculatus* performs best at a P:C ratio near 1:2 [56]. Mineral elements also play indispensable roles: calcium and phosphorus strengthen the exoskeleton, while magnesium and potassium support nerve function and enzymatic activity. Trace minerals like zinc, sodium, and manganese are crucial for immune response and oxidative stress management [57].

Crickets exhibit nutrient-balancing behavior, adjusting their intake to meet species-specific needs [58]. Therefore, feed formulation should consider both nutrient availability and the species’ nutritional ecology. In our study, optimal growth was observed with a balanced intake of protein (~215 g/kg DM), carbohydrates (~400 g/kg DM), and minerals. Crickets fed on the Mixed Weed–Bran Diet, with moderate protein, substantial carbohydrates, and high mineral content (e.g., Fe: 881.4 mg/kg DM; Zn: 64.2 mg/kg DM; Na: 2399.9 mg/kg DM), showed the shortest development time and greatest biomass gain, aligning with previous findings [59,60,61,62,63].

Conversely, diets with extreme levels of protein (>250 or <145 g/kg DM) or carbohydrates (>535 or <277 g/kg DM) delayed development and reduced growth efficiency. For example, the Cassava–Sugar Diet, with its high protein content (250 g/kg DM), resulted in significantly lower survival, indicating potential toxicity or metabolic inefficiency—consistent with earlier studies [12,56].

Survivorship was highest on the Mixed Weed–Bran Diet (88.1%), followed closely by the chicken feed control (85.7%), reinforcing the importance of balanced nutrition. These outcomes align with prior work indicating that protein- and mineral-rich diets improve survival and growth in *A. domesticus* and *G. madagascarensis* [25,36,58,59,60]. Furthermore, crickets on the Mixed Weed–Bran Diet exhibited improved feed conversion ratios (FCRs), yielding larger body sizes and higher biomass, consistent with previous research [8,25,31].

Crude protein and fat contents of the crickets ranged from 51.5 to 64.4 g/100 g DM and 7.4 to 19.1 g/100 g DM, respectively. The highest values were observed in crickets fed the Mixed Weed–Bran and Morning Glory–Cassava Diets—comparable to commercial feed-fed crickets [29]. Diets high in carbohydrates (e.g., Wheat–Bran and Maize–Cassava) led to higher fat accumulation, suggesting conversion of excess carbohydrates into lipids, a trend supported by other studies [58,59,60,61,62,63].

Mineral composition also varied across diets. Crickets fed the Mixed Weed–Bran Diet had the highest concentrations of Fe, Zn, Na, and Ca, while those on the Maize–Cassava Diet had the lowest. These findings are critical not only for insect health but also for human nutrition, as crickets can supply essential minerals such as iron, zinc, magnesium, and calcium—all vital for functions like hemoglobin formation, bone health, and nerve signaling [63,64]. The mineral content of the crickets in this study met or exceeded recommended daily intake values for humans, supporting their use as a nutritious food source [11,65,66,67,68,69,70].

Fatty acid composition was also diet-dependent. Crickets fed the Morning Glory–Bean Diet showed the highest levels of beneficial unsaturated fatty acids (oleic, linoleic, palmitic), known for supporting cardiovascular and neurological health [71,72,73,74,75]. In contrast, the Morning Glory–Cowpea Diet produced the highest saturated fatty acid levels. Importantly, the PUFA/SFA ratios in these diets remained within recommended limits, reinforcing crickets’ potential as a health-promoting food [76,77].

Overall, this study demonstrates that diets made from weeds and agricultural by-products can sustainably support the growth and nutritional quality of *G. sigillatus*. These diets offer a low-risk, cost-effective alternative to human food waste, with fewer safety and regulatory challenges. Agricultural by-products and weeds are generally plant-based, easier to trace, and present minimal contamination risks when properly sourced [12,13,61].

These findings align well with circular economy principles by promoting the valorization of underutilized biomass, especially in resource-rich regions like Madagascar. Given their low cost, availability, and regulatory advantages, such diets are well-suited for scaling up cricket farming. With appropriate documentation, they can potentially meet international food safety standards.

The Mixed Weed–Bran Diet (Optimal) is particularly practical for small-scale farmers in Madagascar, as the ingredients are readily accessible from farms and markets. Farmers are encouraged to harvest and preserve surplus materials during peak seasons for year-round use. Further research should focus on refining diet formulations to optimize growth and nutritional output, particularly examining mineral bioavailability and their effects on human health.

## 5. Conclusions

This study demonstrates that diets developed from weeds and agro by-products can be effectively used as feed for tropical house crickets, providing a sustainable solution for managing agro-waste while supporting a growing food industry. Among the various diets tested, the Mixed Weed–Bran Diet (Optimal) and the Morning Glory–Cassava Diet were the most optimal for cricket growth, yielding nutrient-dense crickets with promising protein, mineral element and essential fatty acid levels. These findings highlight the potential of farmed crickets as an alternative source of protein, mineral elements and essential fatty acids for human consumption, which could reduce dependence on conventional protein sources such as beef, chicken, fish, and soymeal. By utilizing agro-waste, tropical house cricket farming supports circular farming practices, where crickets not only consume weeds and by-products but also produce frass, a valuable organic fertilizer for plants. We therefore recommend large-scale trials to assess the efficiency of these diets in commercial cricket farming, further promoting circular farming and environmental sustainability in food production.

## Figures and Tables

**Table 1:** Ingredients of the experimental and control diets.

Diets	Diet Ingredients	Quantity g/Kg DM
Chicken Feed (Control)	Control	Control
Cassava–Sugar Diet	Cassava leaves, sugar and baking powder	980 g + 10 g + 10 g
Desmodium–Bran Diet	Silver leaf Desmodium, wheat bran, maize bran, and cowpea bran, SWMC	200 g + 200 g + 200 g + 400 g
Morning Glory–Bean Diet	Tropical African morning glory, navy bean bran, maize bran, maize germ, rice bran	300 g + 300 g + 200 g + 100 g + 100 g
Morning Glory–Cassava Diet	Tropical African morning glory, cassava leaves, maize bran, rice bran, cowpea bran, navy bean bran	200 g + 200 g + 200 g + 100 g + 100 g + 200 g
Morning Glory–Cowpea Diet	Tropical African morning glory, cowpea bran, cassava leaf powder, cowpea bran and navy bean bran	300 g + 200 g + 400 g + 100 g
Mixed Weed–Bran Diet (Optimal)	Tropical African morning glory, gallant soldier, cassava leaves, cowpea bran, navy bean bran, maize bran, wheat bran	200 g + 200 g + 150 g + 200 g + 100 g + 100 g + 50 g
Cassava–Gallant Soldier Diet	Cassava leaves, gallant soldier, cowpea bran, tropical African morning glory, taro leaves	300 g + 300 g + 200 g + 100 g + 100 g
Wheat–Bran Diet	Wheat bran and powder	990 g + 10 g
Maize–Cassava Diet	Maize bran, cassava tuber bran, wheat bran, baking powder	330 g + 330 g + 330 g + 10 g

Note: Short names are based on the dominant ingredient.

**Table 2:** Chemical composition of experimental diets and a control diet.

Diet	DM	g/kg DM						Gross Energy (Kcal/kg)
		Moisture	Protein	Fat	Ash	Fiber	Carbohydrate	
Chicken Feed (Control)	906.70 ± 1.20	93.30 ± 1.20	215.00 ± 5.68	29.30 ± 0.58	65.00 ± 2.14	66.20 ± 0.28	531.20 ± 7.10	3248.70 ± 16.30
Cassava–Sugar Diet	905.00 ± 0.95	95.00 ± 0.95	250.00 ± 1.78	34.00 ± 0.82	53.00 ± 1.40	100.00 ± 1.03	468.00 ± 4.80	3178.00 ± 14.82
Desmodium–Bran Diet	871.00 ± 0.36	129.00 ± 0.36	245.00 ± 11.00	38.00 ± 0.91	84.00 ± 0.74	74.00 ± 1.65	430.00 ± 10.74	3042.00 ± 7.50
Morning Glory–Bean Diet	928.00 ± 3.96	72.00 ± 3.96	240.00 ± 0.06	71.00 ± 4.70	101.00 ± 2.70	88.00 ± 1.43	428.00 ± 9.58	3311.00 ± 12.07
Morning Glory–Cassava diet	884.00 ± 1.00	116.00 ± 1.0	235.00 ± 10.26	21.00 ± 0.80	101.00 ± 0.80	106.00 ± 2.97	421.00 ± 12.81	2813.00 ± 13.80
Morning Glory–Cowpea Diet	905.00 ± 3.04	95.00 ± 3.04	225.00 ± 10.51	19.00 ± 0.26	64.00 ± 0.71	62.00 ± 0.44	535.00 ± 13.55	3211.00 ± 10.73
Mixed Weed–Bran Diet (Optimal)	921.00 ± 1.99	79.00 ± 1.99	215.00 ± 4.97	63.00 ± 2.70	118.00 ± 0.79	125.00 ± 1.40	400.00 ± 4.43	3028.00 ± 20.77
Cassava–Gallant Soldier Diet	912.00 ± 0.40	88.00 ± 0.40	200.00 ± 10.06	53.00 ± 0.36	113.00 ± 1.05	128.00 ± 4.00	418.00 ± 11.99	2949.00 ± 14.60
Wheat–Bran Diet	901.00 ± 2.44	99.00 ± 2.14	145.0 ± 9.19	34.000 ± 0.55	43.00 ± 0.75	99.80 ± 3.69	569.00 ± 53.32	3162.00 ± 22.68
Maize–Cassava Diet	880.00 ± 4.04	120.00 ± 4.04	135.0 ± 2.53	13.0 ± 0.33	52.00 ± 0.47	114.00 ± 4.71	566.00 ± 9.48	2021.00 ± 35.03

The results are expressed as means ± SD based on triplicate determinations of dry matter and other proximate components.

**Table 3:** Micro- and macro-mineral values in control diet and experimental diets.

Diet	mg/kg DM					g/kg DM			
	Fe	Cu	Zn	Mn	Na	Mg	Ca	P	K
Chicken Feed	228.10 ± 10.74	16.20 ± 0.47	67.00 ± 1.18	63.90 ± 5.81	1065.40 ± 38.75	2.00 ± 0.01	5.00 ± 0.01	2.00 ± 0.01	13.00 ± 0.01
Cassava–Sugar Diet	61.10 ± 2.64	5.30 ± 0.01	49.50 ± 0.66	73.90 ± 0.48	100.00 ± 2.46	0.10 ± 0.01	0.10 ± 0.01	0.20 ± 0.01	13.00 ± 0.02
Desmodium–Bran Diet	171.10 ± 0.60	6.80 ± 0.63	37.10 ± 2.76	35.90 ± 4.94	1939.30 ± 24.36	2.00 ± 0.01	9.00 ± 0.02	2.00 ± 0.01	12.00 ± 0.01
Morning Glory–Bean Diet	327.20 ± 4.35	18.00 ± 0.74	62.50 ± 1.20	52.80 ± 3.67	1935.00 ± 15.95	3.00 ± 0.01	9.00 ± 0.01	2.00 ± 0.01	17.00 ± 0.02
Morning Glory–Cassava Diet	288.20 ± 64.85	7.30 ± 0.09	45.00 ± 1.53	57.60 ± 0.79	2033.40 ± 34.85	2.00 ± 0.01	7.00 ± 0.01	3.00 ± 0.01	13.00 ± 0.01
Morning Glory–Cowpea Diet	212.50 ± 1.96	19.70 ± 0.05	28.80 ± 0.78	10.20 ± 0.17	1040.80 ± 1.31	2.00 ± 0.01	10.0 ± 0.02	2.00 ± 0.01	12.00 ± 0.02
Mixed Weed–Bran Diet	881.40 ± 19.10	9.70 ± 1.37	64.20 ± 1.03	79.30 ± 0.59	2399.90 ± 58.98	3.00 ± 0.01	11.00 ± 0.01	2.00 ± 0.01	16.00 ± 0.02
Cassava–Gallant Soldier Diet	172.60 ± 12.26	8.60 ± 0.65	35.80 ± 0.61	52.80 ± 2.59	1935.00 ± 123.85	2.00 ± 0.01	6.00 ± 0.01	2.00 ± 0.01	10.00 ± 0.01
Wheat–Bran Diet	46.20 ± 4.35	5.37 ± 0.02	57.90 ± 1.96	83.60 ± 0.54	21.30 ± 0.15	3.00 ± 0.01	1.00 ± 0.01	3.00 ± 0.01	12.00 ± 0.01
Maize–Cassava Diet	38.40 ± 19.15	3.50 ± 0.09	18.90 ± 0.98	85.20 ± 0.35	19.30 ± 0.15	3.00 ± 0.01	1.00 ± 0.01	0.20 ± 0.01	15.00 ± 0.02

The results are expressed as means ± SD based on triplicate determinations of dry matter and other proximate components.

**Table 4:** Proximate values (g/100 g DM) and gross energy (kcal/kg) in *Gryllodes sigillatus* crickets fed on different developed diets and chicken feed.

*G. sigillatus* Reared On	DM	Fat	Protein	Crude Fiber	Ash	Carbohydrates	Gross Energy (kcal/kg)
Chicken Feed (Control)	96.29 ± 0.02 ^a^	12.83 ± 0.092 ^d^	61.08 ± 0.35 ^bc^	6.18 ± 0.083 ^cd^	6.63 ± 0.025 ^c^	9.57 ± 0.417 ^bc^	3981.03 ± 6.11 ^b^
Cassava–Sugar Diet	92.41 ± 0.16 ^e^	7.44 ± 0.56 ^e^	62.79 ± 0.56 ^ab^	10.34 ± 0.14 ^a^	6.92 ± 0.084 ^b^	4.92 ± 0.453 ^de^	3388.00 ± 22.61 ^d^
Desmodium–Bran Diet	95.97 ± 0.29 ^ab^	16.65 ± 0.85 ^bc^	59.38 ± 0.35 ^cd^	5.53 ± 0.28 ^d^	6.79 ± 0.100 ^bc^	7.63 ± 0.902 ^bcd^	4164.93 ± 63.89 ^a^
Morning Glory–Bean Diet	95.61 ± 0.27 ^b^	15.32 ± 1.01 ^bc^	60.89 ± 1.61 ^bc^	5.72 ± 1.74 ^d^	5.20 ± 0.034 ^e^	8.49 ± 2.767 ^bc^	4154.13 ± 94.07 ^a^
Morning Glory–Cowpea Diet	94.51 ± 0.58 ^cd^	13.14 ± 0.41 ^d^	64.39 ± 2.10 ^a^	8.07 ± 0.41 ^b^	6.04 ± 0.032 ^d^	2.86 ± 1.952 ^ef^	3872.70 ± 41.96 ^c^
Morning Glory–Cowpea Diet	95.02 ± 0.17 ^c^	16.17 ± 2.29 ^bc^	52.90 ± 0.59 ^f^	6.37 ± 0.19 ^cd^	4.67 ± 0.205 ^f^	14.91 ± 1.833 ^a^	4167.56 ± 119.97 ^a^
Mixed Weed–Bran Diet (Optimal)	96.41 ± 0.12 ^a^	12.52 ± 0.79 ^d^	64.44 ± 1.61 ^a^	3.36 ± 0.25 ^e^	5.14 ± 0.098 ^e^	10.94 ± 0.792 ^b^	4241.97 ± 41.61 ^a^
Cassava–Gallant Soldier Diet	96.02 ± 0.16 ^ab^	16.66 ± 0.48 ^bc^	58.30 ± 0.48 ^d^	7.72 ± 0.06 ^bc^	6.24 ± 0.078 ^d^	7.09 ± 0.189 ^cd^	4115.43 ± 27.47 ^a^
Wheat–Bran Diet	94.76 ± 0.35 ^cd^	17.71 ± 1.17 ^ab^	56.17 ± 0.14 ^e^	6.94 ± 0.80 ^bcd^	4.43 ± 0.115 ^g^	9.50 ± 1.882 ^bc^	4220.67 ± 54.08 ^a^
Maize–Cassava Diet	94.25 ± 0.12 ^d^	19.06 ± 0.43 ^a^	51.52 ± 0.12 ^f^	6.59 ± 0.24 ^cd^	15.41 ± 0.251 ^a^	1.59 ± 0.261 ^f^	3839.93 ± 32.44 ^c^
F_9,20_	62.38	32.48	51.24	24.43	1956.00	22.77	56.61
*p*	<0.001	<0.001	<0.001	<0.001	<0.001	<0.001	<0.001

The results are expressed as means ± SD based on triplicate determinations of dry matter and other proximate components. Different superscripts in a column indicate significant differences as calculated using the Student–Newman–Keuls test (*p* < 0.05).

## Data Availability

The original contributions presented in this study are included in the article. Further inquiries can be directed to the corresponding author.

## Appendix A

**Table A1:** Mineral elements in *Gryllodes sigillatus* crickets reared on different developed diets and chicken feed.

*G. sigillatus* Reared on	Minerals (mg/100 g DM)								
	Mg	Fe	Ca	Cu	P	Zn	K	Mn	Na
Chicken Feed (Control)	126.04 ± 4.81 ^f^	18.03 ± 0.76 ^b^	407.35 ± 18.98 ^c^	0.21 ± 0.04 ^f^	1151.82 ± 37.71 ^bc^	27.86 ± 1.57 ^b^	1575.41 ± 52.02 ^a^	4.83 ± 016 ^h^	369.89 ± 7.67 ^a^
Cassava–Sugar Diet	172.11 ± 0.96 ^a^	14.62 ± 0.31 ^c^	369.48 ± 18.92 ^d^	4.47 ± 0.03 ^a^	1127.05 ± 36.85 ^bcd^	24.01 ± 0.34 ^c^	1282.59 ± 9.42 ^ab^	10.01 ± 0.19 ^a^	214.93 ± 28.13 ^b^
Desmodium–Bran Diet	142.40 ± 5.13 ^d^	10.53 ± 1.56 ^d^	436.47 ± 9.06 ^c^	2.58 ± 0.05 ^e^	967.70 ± 45.04 ^e^	19.44 ± 0.72 ^d^	1413.74 ± 23.83 ^a^	4.86 ± 0.20 ^h^	221.02 ± 29.47 ^b^
Morning Glory–Bean Diet	144.58 ± 7.52 ^d^	12.86 ± 1.28 ^cd^	320.15 ± 20.40 ^e^	4.05 ± 0.11 ^b^	1026.38 ± 6.11 ^cde^	24.09 ± 0.48 ^c^	1328.03 ± 3.05 ^ab^	7.22 ± 0.31 ^d^	187.64 ± 1.56 ^bc^
Morning Glory–Cassava Diet	134.57 ± 3.37 ^e^	12.74 ± 0.61 ^cd^	534.41 ± 10.00 ^b^	3.55 ± 0.00 ^c^	1166.03 ± 21.58 ^b^	23.52 ± 0.12 ^c^	1580.65 ± 5.39 ^a^	5.45 ± 0.04 ^g^	187.93 ± 8.26 ^bc^
Morning Glory–Cowpea Diet	151.17 ± 1.16 ^c^	13.49 ± 3.45 ^cd^	373.52 ± 6.48 ^d^	2.83 ± 0.03 ^d^	1004.47 ± 10.59 ^de^	23.49 ± 0.66 ^c^	1230.80 ± 3.20 ^ab^	7.71 ± 0.27 ^c^	210.52 ± 28.12 ^b^
Mixed Weed–Bran Diet (Optimal)	143.33 ± 1.37 ^d^	23.86 ± 1.28 ^a^	839.05 ± 47.02 ^a^	4.49 ± 0.17 ^a^	1495.67 ± 159.81 ^a^	34.36 ± 1.31 ^a^	1607.36 ± 6.09 ^a^	6.82 ± 0.17 ^e^	370.48 ± 4.64 ^a^
Cassava–Gallant Soldier Diet	164.80 ± 2.48 ^b^	15.61 ± 0.05 ^bc^	370.54 ± 0.70 ^d^	4.27 ± 0.04 ^a^	1050.08 ± 3.20 ^bcde^	26.72 ± 0.59 ^b^	1207.10 ± 5.27 ^ab^	8.85 ± 0.02 ^b^	194.30 ± 26.95 ^bc^
Wheat–Bran Diet	144.55 ± 2.71 ^d^	10.81 ± 0.12 ^d^	183.59 ± 3.11 ^g^	3.55 ± 0.15 ^c^	934.73 ± 12.46 ^e^	22.81 ± 0.37 ^c^	1098.59 ± 14.40 ^ab^	6.19 ± 0.09 ^f^	114.89 ± 11.67 ^d^
Maize–Cassava Diet	95.06 ± 2.11 ^g^	15.85 ± 1.22 ^bc^	266.53 ± 10.10 ^f^	3.04 ± 0.30 ^d^	724.68 ± 25.27 ^f^	19.59 ± 0.20 ^d^	894.23 ± 0.05 ^b^	6.62 ± 0.45 ^e^	159.68 ± 4.79 ^c^
F_9,20_	96.50	24.79	257.70	309.8	37.22	93.30	40.70	163.40	59.79
*p*	<0.001	<0.001	<0.001	<0.001	<0.001	<0.001	<0.001	<0.001	<0.001

The results are expressed as means ± SD based on triplicate determinations of dry matter and other proximate components. Different superscripts in a column indicate significant differences, Student–Newman–Keuls test (*p* < 0.05).

**Table A2:** Fatty acid content (%) of oils extracted from *Gryllodes sigillatus* crickets fed on different developed diets and chicken feed.

Fatty Acid	Chicket Feed (Control)	Cassava–Sugar Diet	Desmodium–Bran Diet	Morning Glory–Bean Bran Diet	Morning Glory–Cassava Diet	Morning Glory–Cowpea Diet	Mixed Weed–Bran Diet (Optimal)	Cassava–Gallant Soldier Diet	Wheat–Bran Diet	Maize–Cassava Diet	F_9,20_	*p*-Value
Saturated fatty acids (SFAs)												
Lauric acid (C12:0)	0.11 ± 0.01 ^cd^	0.22 ± 0.01 ^bc^	0.13 ± 0.02 ^cd^	0.90 ± 0.13 ^a^	0.11 ± 0.01 ^cd^	0.25 ± 0.02 ^b^	0.19 ± 0.01 ^bc^	0.07 ± 0.01 ^d^	0.11 ± 0.01 ^cd^	0.22 ± 0.01 ^bc^	92.86	0.001
Myristic acid (C14:00	1.79 ± 0.04 ^c^	1.49 ± 0.11 ^cd^	1.52 ± 0.14 ^cd^	1.32 ± 0.09 ^d^	1.23 ± 0.07 ^d^	3.96 ± 0.28 ^a^	1.81 ± 0.15 ^c^	1.22 ± 0.02 ^d^	1.54 ± 0.19 ^cd^	2.60 ± 0.06 ^b^	57.37	0.001
Pentadecanoic acid (C15:0)	0.19 ± 0.01 ^e^	0.66 ± 0.04 ^a^	0.38 ± 0.04 ^bc^	0.33 ± 0.01 ^cd^	0.26 ± 0.02 ^de^	0.69 ± 0.07 ^a^	0.39 ± 0.05 ^bc^	0.34 ± 0.01 ^cd^	0.34 ± 0.09 ^cd^	0.46 ± 0.03 ^b^	37.07	0.001
Palmitic acid (C16:0)	26.59 ± 0.70 ^c^	21.16 ± 1.00 ^de^	20.24 ± 1.29 ^e^	0.49 ± 0.13 ^g^	20.34 ± 0.20 ^e^	37.06 ± 1.34 ^a^	23.86 ± 2.74 ^d^	15.73 ± 0.78 ^f^	27.38 ± 2.19 ^c^	32.93 ± 0.79 ^b^	166.70	0.001
Margaric acid (C17:0)	0.65 ± 0.04 ^d^	1.79 ± 0.17 ^b^	0.97 ± 0.20 ^d^	0.74 ± 0.18 ^d^	0.66 ± 0.06 ^d^	1.45 ± 0.15 ^c^	0.86 ± 0.18 ^d^	0.79 ± 0.01 ^d^	0.85 ± 0.21 ^d^	2.10 ± 0.06 ^a^	37.39	0.001
Stearic acid (C18:0)	16.25 ± 0.52 ^bc^	15.47 ± 1.14 ^bc^	8.90 ± 0.28 ^d^	13.10 ± 1.97 ^c^	9.89 ± 0.84 ^d^	18.48 ± 1.79 ^a^	10.73 ± 0.92 ^d^	10.76 ± 0.82 ^d^	13.42 ± 1.40 ^c^	16.66 ± 0.91 ^ab^	22.01	0.001
Arachidic acid (C20:0)	2.01 ± 0.26 ^bcd^	2.12 ± 0.22 ^bcde^	1.33 ± 0.31 ^cd^	3.53 ± 0.31 ^a^	1.81 ± 0.19 ^bcd^	2.56 ± 0.24 ^b^	1.11 ± 0.08 ^d^	1.14 ± 0.30 ^d^	2.33 ± 0.88 ^bc^	2.60 ± 0.78 ^b^	9.09	0.001
Behenic acid (C22:0)	0.26 ± 0.02 ^d^	0.96 ± 0.25 ^b^	0.54 ± 0.08 ^cd^	1.47 ± 0.20 ^a^	0.45 ± 0.04 ^cd^	1.04 ± 0.15 ^b^	0.61 ± 0.12 ^c^	0.38 ± 0.02 ^cd^	0.33 ± 0.08 ^cd^	0.34 ± 0.08 ^cd^	28.36	0.001
Lignoceric acid (C24:0)	nd	nd	nd	nd	nd	nd	nd	0.35 ± 0.00	nd	nd	nd	0.001
Polyunsaturated fatty acid (PUFAs)												
Alpha-linolenic acid (C 18:3(n-3))	0.82 ± 0.01 ^e^	1.24 ± 0.08 ^c^	1.25 ± 0.14 ^c^	0.84 ± 0.10 ^e^	1.44 ± 0.06 ^ab^	1.35 ± 0.05 ^abc^	1.30 ± 0.09 ^bc^	1.50 ± 0.04 ^a^	1.02 ± 0.07 ^d^	0.46 ± 0.01 ^f^	57.89	0.001
Linoleic acid (C18:2(n-6))	16.38 ± 0.10 ^e^	24.73 ± 1.58 ^c^	25.11 ± 2.85 ^c^	16.96 ± 2.07 ^e^	29.01 ± 1.22 ^ab^	27.19 ± 1.02 ^abc^	26.12 ± 1.71 ^bc^	30.11 ± 0.64 ^a^	20.55 ± 1.36 ^d^	9.23 ± 0.21 ^f^	58.02	0.001
Monounsaturated fatty acids (MUFAs)												
Oleic acid (C18:1(n-9))	34.95 ± 1.45 ^bcd^	30.16 ± 2.00 ^d^	39.63 ± 4.52 ^b^	60.32 ± 0.13 ^a^	34.80 ± 2.29 ^bcd^	5.97 ± 0.39 ^e^	33.02 ± 3.08 ^cd^	37.58 ± 2.48 ^bc^	32.13 ± 2.30 ^cd^	32.40 ± 0.58 ^cd^	96.75	0.001
MUFAs	34.95 ± 1.45 ^bcd^	30.15 ± 2.00 ^d^	39.63 ± 4.52 ^b^	60.32 ± 0.13 ^a^	34.80 ± 2.29 ^bcd^	5.97 ± 0.39 ^e^	33.02 ± 3.08 ^cd^	37.58 ± 2.48 ^bc^	32.13 ± 2.30 ^cd^	32.40 ± 0.58 ^cd^	96.75	0.001
PUFAs	17.20 ± 0.11 ^e^	25.97 ± 1.66 ^c^	26.36 ± 2.99 ^c^	17.81 ± 2.18 ^e^	30.45 ± 1.28 ^ab^	28.54 ± 1.07 ^abc^	27.42 ± 1.80 ^bc^	31.61 ± 0.68 ^a^	21.57 ± 1.43 ^d^	9.69 ± 0.22 ^f^	58.01	0.001
SFAs	47.85 ± 1.37 ^c^	43.88 ± 0.51 ^c^	34.01 ± 1.69 ^e^	21.88 ± 2.07 ^f^	34.75 ± 1.20 ^e^	65.49 ± 1.37 ^a^	39.56 ± 3.19 ^d^	30.81 ± 1.84 ^e^	46.30 ± 3.72 ^c^	57.91 ± 0.76 ^b^	124.30	0.001
EFAs	17.20 ± 0.11 ^e^	25.97 ± 1.66 ^c^	26.36 ± 2.99 ^c^	17.80 ± 2.18 ^e^	30.45 ± 1.28 ^ab^	28.54 ± 1.07 ^abc^	27.42 ± 1.80 ^bc^	31.61 ± 0.68 ^a^	21.57 ± 1.43 ^d^	9.69 ± 0.22 ^f^	58.01	0.001
PUFA/SFA	0.36	0.59	0.78	0.81	0.88	0.44	0.69	1.02	0.47	0.17		

The results are expressed as means ± SD based on triplicate determinations of dry matter and other proximate components. Different superscripts in a row indicate significant differences, Student–Newman–Keuls test (*p* < 0.05).

## References

[1] United Nations (2023). World Population Prospects. Summary of Results.

[2] Behixhe A., Irene B., Gloriana C., Silvia I., Anna A., Federico G., Matteo F., Francesca T., Tommaso P., Cristina T. (2025). Modulating the nutritional value of *Acheta domesticus* (house cricket) through the eco-sustainable *Ascophyllum nodosum* dietary supplementation. J. Food Compos. Anal..

[3] Tomlinson I. (2013). Doubling food production to feed the 9 billion: A critical perspective on a key discourse of food security in the UK. J. Rural Stud..

[4] FAO, Global Forum for food and agriculture (GFFA) (2024). Food Systems for Our Future: Joining Forces for a Zero Hunger World, Berlin, Germany, 17-20/01/2024.

[5] Nasir S.Q., Palupi E., Nasution Z., Ploeger A., Susanto I., Setiawan B., Rimbawan R., Jayanegara A. (2024). Edible insects as a sustainable protein source: A meta-analysis. J. Insects Food Feed.

[6] O’Sullivan J.N. (2020). The social and environmental influences of population growth rate and demographic pressure deserve greater attention in ecological economics. Ecol. Econ..

[7] Zandi-Sohani N., Tomberlin J.K. (2024). Comparison of growth and composition of black soldier fly (*Hermetia illucens* L.) larvae reared on sugarcane by-products and other substrates. Insects.

[8] Magara H.J.O., Solofondranohatra C.L., Hugel S., Fisher B.L. (2025). Weeds and agro byproducts for sustainable farming of edible field cricket, *Gryllus madagascarensis* (Orthoptera: Gryllidae). PLoS ONE.

[9] Lange K.W., Nakamura Y. (2021). Edible insects as future food: Chances and challenges. J. Future Foods.

[10] Murugu D.K., Onyango A.N., Ndiritu A.K., Osuga I.M., Xavier C., Nakimbugwe D., Tanga C.M. (2021). From farm to fork: Crickets as alternative source of protein, minerals, and vitamins. Front. Nutr..

[11] Magara H.J.O., Niassy S., Ayieko M.A., Mukundamago M., Egonyu J.P., Tanga C.M., Kimathi E.K., Ongere J.O., Fiaboe K.K.M., Hugel S. (2021). Edible Crickets (Orthoptera) Around the world: Distribution, nutritional value, and other benefits—A review. Front. Nutr..

[12] Magara H.J.O., Hugel S., Fisher B.L. (2024). Effect of feed on the growth performance, nutrition content and cost of raising the field cricket (*Gryllus madagascarensis*) as a sustainable nutrient source in Madagascar. Foods.

[13] Oonincx D.G., van Broekhoven S., van Huis A., van Loon J.J. (2015). Feed conversion, survival and development, and composition of four insect species on diets composed of food by-products. PLoS ONE.

[14] Kuo C., Fisher B.L. (2022). A literature review of the use of weeds and agricultural and food industry by-products to feed farmed crickets (Insecta; Orthoptera; Gryllidae). Front. Sustain. Food Syst..

[15] Orinda M.A., Oloo J., Magara H.J.O., Ekesi S., Nanna R., Ayieko M. (2021). Cricket Rearing Handbook.

[16] Magara H.J.O., Tanga C.M., Fisher B.L., Azrag A.G.A., Niassy S., Egonyu J.P., Hugel S., Roos N., Ayieko M.A., Sevgan S. (2024). Impact of temperature on the bionomics and geographical range margins of the two-spotted field cricket *Gryllus bimaculatus* in the world: Implications for its mass farming. PLoS ONE.

[17] Andrianorosoa Ony C., Solofondranohatra C.L., Ramiadantsoa T., Ravelomanana A., Ramanampamonjy R.N., Hugel S. (2024). Effect of cricket frass fertilizer on growth and pod production of green beans (*Phaseolus vulgaris* L.). PLoS ONE.

[18] Baiano A. (2020). Edible insects: An overview on nutritional characteristics, safety, farming, production technologies, regulatory framework, and socio-economic and ethical implications. Trends Food Sci. Technol..

[19] Magara H.J.O. (2020). Assessment of Cricket Species Composition, Feed Substrates, Optimal Temperature and Nutritional Content of Edible Cricket *Scapsipedus icipe*. Doctoral Dissertation.

[20] Fisher B., Hugel S. (2022). Edible insects traditions and uses on Madagascar. The New Natural History of Madagascar.

[21] Jongema Y. (2017). List of Edible Insects of the World.

[22] Govorushko S. (2019). Global status of insects as food and feed source: A review. Trends Food Sci. Technol..

[23] Tanga C.M., Egonyu J.P., Beesigamukama D., Niassy S., Emily K., Magara H.J.O., Omuse E.R., Subramanian S., Ekesi S. (2021). Edible insect farming as an emerging and profitable enterprise in East Africa. Curr. Opin. Insect. Sci..

[24] Van Itterbeeck J., Pelozuelo L. (2022). How many edible insect species are there? A not so simple question. Diversity.

[25] Rowe E., López K.Y., Robinson K.M., Baudier K.M., Barrett M. (2024). Farmed cricket (*Acheta domesticus*, *Gryllus assimilis*, and *Gryllodes sigillatus*; Orthoptera) welfare considerations: Recommendations for improving global practice. J. Insects Food Feed..

[26] Walker T.J. (2011). Tropical house cricket, *Gryllodes sigillatus* (F. Walker 1869). Singing Insects of North America.

[27] Bertola M., Mutinelli F. (2021). A Systematic Review on Viruses in Mass-Reared Edible Insect Species. Viruses.

[28] Krönauer T., Döring T.F., Röder G. (2025). Advances in production of crickets as food and animal feed: A case study of five wild European plants as cricket feed. Sustain. Agric. Res..

[29] Yuzer A., Dizhi X., Retno T.A., Joey W., Le W. (2022). Insects as a feed ingredient for fish culture: Status and trends. Aquac. Fish..

[30] Muzzatti M.J., McConnell E., Neave S., MacMillan H.A., Bertram S.M. (2022). Fruitful female fecundity after feeding *Gryllodes sigillatus* (Orthoptera: Gryllidae) royal jelly. Can. Entomol..

[31] Kasdorf S.Y., Muzzatti M.J., Haider F., Bertram S.M., MacMillan H.A. (2025). Brewery waste as a sustainable protein source for the banded cricket (*Gryllodes sigillatus*). J. Insects Food Feed.

[32] Comunian T.A., Silva M.P., Souza C.J.F. (2021). The use of food by-products as a novel for functional foods: Their use as ingredients and for the encapsulation process. Trends Food Sci. Technol..

[33] Magara H.J.O., Tanga C.M., Ayieko M.A., Hugel S., Mohamed S.A., Khamis F.M., Salifu D., Niassy S., Sevgan S., Fiaboe K.K.M. (2019). Performance of newly described native edible cricket *Scapsipedus icipe* (Orthoptera: Gryllidae) on various diets of relevance for farming. J. Econ. Entomol..

[34] Vastolo A., Calabrò S., Cutrignelli M.I. (2022). A review on the use of agro-industrial CO-products in animals’ diets. Ital. J. Anim. Sci..

[35] Otieno M.H.J., Ayieko M.A., Niassy S., Salifu D., Abdelmutalab A.G., Fathiya K.M., Subramanian S., Fiaboe K.K.M., Roos N., Ekesi S. (2019). Integrating temperature-dependent life table data into Insect Life Cycle Model for predicting the potential distribution of *Scapsipedus icipe* Hugel & Tanga. PLoS ONE.

[36] Sorjonen J.M., Valtonen A., Hirvisalo E., Karhapää M., Lehtovaara V.J., Lindgren J., Marnila P., Mooney P., Mäki M., Siljander-Rasi H. (2019). The plant-based by-product diets for the mass-rearing of *Acheta domesticus* and *Gryllus bimaculatus*. PLoS ONE.

[37] Bawa M., Songsermpong S., Kaewtapee C., Chanput W. (2020). Effect of diet on the growth performance, feed conversion, and nutrient content of the house cricket. J. Insect Sci..

[38] Sorjonen J.M., Karhapa M., Valtonen A., Holm A., Roininen H. (2021). Performance of the house cricket (*Acheta domesticus*) on by-product diets in small-scale production. J. Insects Food Feed.

[39] Finke M.D., DeFoliart G.R., Benevenga N.J. (1989). Use of a four-parameter logistic model to evaluate the quality of the protein from three insect species when fed to rats. J. Nutr..

[40] AOAC (2016). International Official Methods of Analysis.

[41] Ajdini B., Biancarosa I., Cardinaletti G., Illuminati S., Annibaldi A., Girolametti F., Truzzi C. (2024). The use of seaweed as sustainable feed ingredient for the house cricket (*Acheta domesticus*): Investigating cricket performance and nutritional composition. J. Insects Food Feed.

[42] AOAC (2010). International Official Methods of Analysis.

[43] Galland-Irmouli A.V., Fleurence J., Lamghari R., Luçon M., Rouxel C., Barbaroux O., Guéant J.L. (1999). Nutritional value of proteins from edible seaweed *Palmaria palmata* (dulse). J. Nutr. Biochem..

[44] Ritvanen T., Pastell H., Welling A., Raatikainen M. (2020). The nitrogen-to-protein conversion factor of two cricket species—*Acheta domesticus* and *Gryllus bimaculatus*. Agric. Food Sci..

[45] Biancarosa I., Espe M., Bruckner C.G., Heesch S., Liland N., Waagbø R., Lock E.J. (2017). Amino acid composition, protein content, and nitrogen-to-protein conversion factors of 21 seaweed species from Norwegian waters. J. Appl. Phycol..

[46] AOAC (Association of Official Analytical Chemists) (1990). Official Methods of Analysis (15th ed.). Off. Methods Anal. J. Assoc. Off. Anal. Chem..

[47] Fairulnizal M.M., Vimala B., Rathi D.N., Naeem M.M., Jian Z., Xichang W. (2019). Atomic absorption spectroscopy for food quality evaluation. Woodhead Publishing Series in Food Science, Technology and Nutrition, Evaluation Technologies for Food Quality.

[48] McKinistry P.J., Indyka H.E., Kim N.D. (1999). The determination of major and minor elements in milk and infant formula by slurry nebulisation and inductively coupled plasma-optical emmission spectrometry (ICP-OES). Food Chem..

[49] Rahman N., Hashem S., Akther S., Jothi J.S. (2023). Impact of various extraction methods on fatty acid profile, physicochemical properties, and nutritional quality index of pangus fish oil. Food Sci. Nutr..

[50] Truzzi C., Giorgini E., Annibaldi A., Antonucci M., Illuminati S., Scarponi G., Riolo P., Isidoro N., Conti C., Zarantoniello M. (2020). Fatty acids profile of black soldier fly (*Hermetia illucens*): Influence of feeding substrate based on coffee-waste silverskin enriched with microalgae. Anim. Feed Sci. Technol..

[51] R version 4.4.2 (Core Team) (2024). R: A Language and Environment for Statistical Computing.

[52] Stabile C., Muzzatti M.J., Haider F., Bertram S.M., MacMillan H.A. (2024). Beyond Growth: The Impact of Diet on Body Composition in the Banded Cricket (*Gryllodes Sigillatus*). A Preprint. https://ssrn.com/abstract=5034863.

[53] Vaga M., Berggren Å., Jansson A. (2021). Growth, survival and development of house crickets (*Acheta domesticus*) fed flowering plants. J. Insects Food Feed.

[54] Hunt A.S., Ward A.M., Ferguson G. (2001). Effects of a High Calcium Diet on Gut Loading in Varying Ages of Crickets (Acheta domesticus) and Mealworms (Tenebrio molitor). https://nagonline.net/wp-content/uploads/2014/02/Hunt-HighCaDiet.pdf.

[55] Peterson T.N., Welti E.A., Kaspari M. (2021). Dietary sodium levels affect grasshopper growth and performance. Ecosphere.

[56] Clissold F.J., Tedder B.J., Conigrave A.D., Simpson S.J. (2010). The Gastrointestinal Tract as a Nutrient-Balancing. Organ. Proc. R. Soc. B.

[57] Finke M.D. (2015). Complete Nutrient Content of Four Species of Feeder Insects. Zoo Biol..

[58] Raubenheimer D., Simpson S.J. (2003). Nutrient Balancing in Grasshoppers: Behavioural and Physiological Correlates of Dietary Breadth. J. Exp. Biol..

[59] Roeder K.A., Behmer S.T. (2014). Lifetime consequences of food protein-carbohydrate content for an insect herbivore. Funct. Ecol..

[60] Dobermann D., Michaelson L., Field L.M. (2018). The effect of an initial high-quality feeding regime on the survival of *Gryllus bimaculatus* (black cricket) on bio-waste. J. Insects Food Feed.

[61] Ajdini A., Peñaranda D.S., Stawarczyk M., Bengtsson J. (2023). Seaweed inclusion reduces fat and alters fatty acid composition in house crickets (*Acheta domesticus*). Front. Nutr..

[62] Tzompa-Sosa D.A., Dewettinck K., Provijn P., Brouwers J.F., Meulenaer d.B., Dennis Oonincx D.G.A.B. (2021). Lipidome of cricket species used as food. Food Chem..

[63] Palupi E., Nasir S.Q., Jayanegara A., Susanto I., Ismail A., Iwansyah A.C., Setiawan B., Sulaeman A., Damanik M.R.M., Filianty F. (2025). Meta-analysis on the fatty acid composition of edible insects as a sustainable food and feed. Future Foods.

[64] Pastell H., Mellberg S., Ritvanen T., Raatikainen M., Mykkänen S., Niemi J., Latomäki I., Wirtanen G. (2021). How does locally produced feed affect the chemical composition of reared house crickets (*Acheta domesticus*)?. ACS Food Sci. Technol..

[65] Siddiqui S.A., Zhao T., Fitriani A., Rahmadhia S.N., Alirezalu K., Fernando I. (2024). *Acheta domesticus* (house cricket) as human foods—An approval of the European commission—A systematic review. Food Front..

[66] (2019). Agricultural Research Service. Usual Nutrient Intake from Food and Beverages, by Gender and Age, What We Eat in America.

[67] Van Peer M., Frooninckx L., Coudron C., Berrens S., Álvarez C., Deruytter D., Verheyen G., Van Miert S., Savoldelli S., Spranghers T. (2021). Insects’ valorisation potential of using organic side streams as feed for *Tenebrio molitor*, *Acheta domesticus*, *Locust migratoria*. Insects.

[68] Jucker C., Belluco S., Oddon S.B., Ricci A., Bonizzi L., Lupi D., Savoldelli S., Biasato I., Caimi C., Mascaretti A. (2022). Impact of some local organic by-products on *Acheta domesticus* growth and meal production. J. Insects Food Feed..

[69] Li Y., Wang S., Liu Y., Lu Y., Zhou M., Wang S., Wang S. (2020). The effect of different dietary sugars on the development and fecundity of *Harmonia axyridis*. Front. Physiol..

[70] Kulma M., Tuůmová V., Fialová A., Kourimská L. (2020). Insect consumption in the Czech Republic: What the eye does not see, the heart does not grieve over. J. Insects Food Feed..

[71] Orkusz A. (2021). Nutritional value and health-promoting properties of edible insects. J. Insects Food Feed.

[72] Rempel J., Grover K., El-Matary W. (2021). Micronutrient deficiencies and anaemia in children with inflammatory bowel disease. Nutrients.

[73] Paul A., Frederich M., Megido R.C., Alabi T., Malik P., Uyttenbroeck R., Francis F., Blecker C., Haubruge E., Lognay G. (2017). Insect fatty acids: A comparison of lipids from three Orthopterans and *Tenebrio molitor* L. larvae. J. Asia Pac. Entomol..

[74] Fombong F., Kinyuru J.N., Ng’ang’a J., Ayieko M.A., Vanden Broeck J., Van Der Borght M. (2021). Affordable processing of edible orthopterans provides a highly nutritive source of food ingredients. Foods.

[75] Kolobe S.D., Manyelo E.M., Malematja E., Sebola N.A., Mabelebele M. (2023). Fats and major fatty acids present in edible insects utilised as food and livestock feed. J. Vet. Sci..

[76] Brown T.J., Brainard J., Song F., Wang X., Abdelhamid A., Hooper L., PUFAH Group (2019). Omega-3, omega-6, and total dietary polyunsaturated fat for prevention and treatment of type 2 *Diabetes mellitus*: Systematic review and meta-analysis of randomised controlled trials. BMJ.

[77] Magara H.J.O., Hugel S., Fisher L.B. (2025). Nutritional composition of four grasshopper species frequently consumed in Madagascar: Insights for nutritional contribution and alternative insect farming. Foods.

